# Chemical and morphological characterization of sugarcane bagasse submitted to a delignification process for enhanced enzymatic digestibility

**DOI:** 10.1186/1754-6834-4-54

**Published:** 2011-11-28

**Authors:** Camila Alves Rezende, Marisa Aparecida de Lima, Priscila Maziero, Eduardo Ribeiro deAzevedo, Wanius Garcia, Igor Polikarpov

**Affiliations:** 1 Instituto de Física de São Carlos, Universidade de São Paulo, Caixa Postal 369, CEP 13560-970, São Carlos, SP, Brazil; 2 Escola de Engenharia de Lorena, Universidade de São Paulo, CEP 12602-810, Lorena, SP, Brazil; 3 Universidade Federal do ABC, CEP 009210-170, Santo André, SP, Brazil

## Abstract

**Background:**

In recent years, biorefining of lignocellulosic biomass to produce multi-products such as ethanol and other biomaterials has become a dynamic research area. Pretreatment technologies that fractionate sugarcane bagasse are essential for the successful use of this feedstock in ethanol production. In this paper, we investigate modifications in the morphology and chemical composition of sugarcane bagasse submitted to a two-step treatment, using diluted acid followed by a delignification process with increasing sodium hydroxide concentrations. Detailed chemical and morphological characterization of the samples after each pretreatment condition, studied by high performance liquid chromatography, solid-state nuclear magnetic resonance, diffuse reflectance Fourier transformed infrared spectroscopy and scanning electron microscopy, is reported, together with sample crystallinity and enzymatic digestibility.

**Results:**

Chemical composition analysis performed on samples obtained after different pretreatment conditions showed that up to 96% and 85% of hemicellulose and lignin fractions, respectively, were removed by this two-step method when sodium hydroxide concentrations of 1% (m/v) or higher were used. The efficient lignin removal resulted in an enhanced hydrolysis yield reaching values around 100%. Considering the cellulose loss due to the pretreatment (maximum of 30%, depending on the process), the total cellulose conversion increases significantly from 22.0% (value for the untreated bagasse) to 72.4%. The delignification process, with consequent increase in the cellulose to lignin ratio, is also clearly observed by nuclear magnetic resonance and diffuse reflectance Fourier transformed infrared spectroscopy experiments. We also demonstrated that the morphological changes contributing to this remarkable improvement occur as a consequence of lignin removal from the sample. Bagasse unstructuring is favored by the loss of cohesion between neighboring cell walls, as well as by changes in the inner cell wall structure, such as damaging, hole formation and loss of mechanical resistance, facilitating liquid and enzyme access to crystalline cellulose.

**Conclusions:**

The results presented herewith show the efficiency of the proposed method for improving the enzymatic digestibility of sugarcane bagasse and provide understanding of the pretreatment action mechanism. Combining the different techniques applied in this work warranted thorough information about the undergoing morphological and chemical changes and was an efficient approach to understand the morphological effects resulting from sample delignification and its influence on the enhanced hydrolysis results.

## Background

Sugarcane is used worldwide as a feedstock for ethanol and sugar production. In Brazil, for instance, *circa *570 million tons of sugarcane were produced in 2009 [1]. After sugarcane is milled for juice extraction, bagasse is obtained as a residue, which corresponds to about 25% of the total weight and contains 60% to 80% of carbohydrates [2]. The fermentation of these carbohydrates could significantly improve bioethanol productivity and sustainability but, instead, bagasse is discarded as agricultural waste or burned for energy supply in sugar and ethanol mills [2-5]. Both alternatives are, however, pollutant and inefficient in making use of the chemical energy available in the biomass [6,7].

Fractionation of bagasse components and their conversion to fermentable sugars is essential in enabling this renewable feedstock to be used for biofuel production [4,8]. Similarly to other plant cell walls, sugarcane bagasse is mainly formed by two carbohydrate fractions (cellulose and hemicellulose) embedded in a lignin matrix. Lignin is a phenolic macromolecule, resistant to enzyme attack and degradation, and thus its content and distribution are recognized as the most important factors determining cell wall recalcitrance to hydrolysis [3,4,9,10].

Pretreatment technologies applied to lignocellulosic substrates are necessary to decrease this recalcitrance and to improve the yields of monomeric fermentable sugars that are liberated by enzymatic hydrolysis [4,11]. Different pretreatment methods have singular action mechanisms. They may decrease cellulose crystallinity and/or the degree of polymerization, increase accessible surface areas or selectively remove hemicellulose and lignin from the lignocellulosic matrix. An effective pretreatment strategy should also minimize carbohydrate degradation and the production of enzyme inhibitors and toxic products for fermenting microorganisms [10,12].

A variety of pretreatments have been applied to different lignocellulosic matrices [8,11]. These include physical processes such as milling [13,14] and irradiation [10,15]; physical-chemical treatments, using hot water and/or steam explosion [9,16], ammonia explosion [17], organic and ionic solvents [18,19], supercritical fluids [20,21], diluted acids and/or bases [2,6,22], sulfite [23,24] and nitrobenzene and copper [25]; and, finally, biological pretreatments using bacteria and fungi [26,27].

Chemical pretreatments using acid are considered effective and economical [28]. Acids hydrolyze hemicellulose and produce a liquid phase rich in xylose, with minor amounts of lignin derivatives. Thus it is an outstanding method for hemicellulose recovery [29,30], and it has been successfully applied to sugarcane bagasse [31,32]. Geddes and collaborators compared sulfuric and phosphoric acid efficiency in dissolving hemicellulose from sugarcane bagasse and obtained similar maximum sugar yields for both acids (257 g of sugar per kilogram of bagasse and 246 g of sugar per kilogram of bagasse, respectively) at a 1% concentration (145°C, 1 h) [31]. High yields of hemicellulose removal (up to 90%) with only 15% of cellulose loss were also obtained by Rocha *et al*. on sugarcane bagasse, using a mixture of sulfuric and acetic acids [32].

Alkali treatments were initially used to increase biomass digestibility for animal feeding. Diluted alkali solutions lead to the disruption of lignocellulosic cell walls by dissolving hemicellulose, lignin and silica, by hydrolyzing uronic and acetic acid esters and by swelling cellulose [3,7]. Lignin decomposition is usually attributed to the cleavage of the α-aryl ether bonds from its polyphenolic monomers, while hemicellulose dissolution and cellulose swelling are a consequence of hydrogen bond weakening [7]. Sodium hydroxide (NaOH) presents the greatest degradation and subsequent fermentation yields when compared to other alkalis, such as sodium carbonate, ammonium hydroxide, calcium hydroxide and hydrogen peroxide [33,34]. Rodríguez-Vázquez *et al*. [33] used a NaOH solution to treat the pith component of sugarcane bagasse (0.2 g of NaOH per pith gram), obtaining a maximum digestibility of 71% at 92°C.

In the present work, a consecutive two-step pretreatment was applied to sugarcane bagasse samples. It included an acid step aimed predominantly at removing hemicellulose, followed by an alkaline treatment with NaOH to remove lignin. A similar procedure was previously applied to sugarcane bagasse by our research group to test the hydrolysis efficiency of different enzymatic cocktails, and hydrolysis yields up to 97% were obtained [35]. In this paper, we describe the morphology and crystallinity of sugarcane bagasse during pretreatment and their relation to chemical composition and to the experimental hydrolysis yields. Although comprehensive chemical characterization of decomposition products have been undertaken for various plant species, very few studies focus on examining their morphology and physical chemistry properties during degradation. This is, however, essential for improving pretreatment strategies and for understanding sample susceptibility under enzyme attack [4,5,36].

## Results

### Chemical composition

The chemical composition of untreated bagasse samples and of samples submitted to acid (sulfuric acid (H_2_SO_4_), 1%) and alkaline pretreatments (NaOH 0.25% to 4%) is presented in Table 1. Percentages of cellulose, hemicellulose, lignin and ashes were calculated on a dry weight basis. Values for cellulose included glucose, cellobiose and hydroxymethylfurfural amounts quantified by HPLC. Hemicellulose comprised xylose, arabinose, furfural, glucuronic and acetic acids, while the total lignin amount was calculated by adding up the concentrations of soluble and insoluble lignins. Ash was the remaining inorganic fraction after the bagasse sample was carbonized in a muffle. Mass closure was obtained by adding cellulose, hemicellulose, lignin and ash percentages for each sample, and the total value obtained is shown in Table 1. Biomass yield after each step is also given in the right column of Table 1.

**Table 1:** Chemical composition of the untreated bagasse sample and samples submitted to acid and alkali pretreatments.

Bagasse samples	Bagasse composition (%)					
	
	Cellulose	Hemicellulose	Lignin	Ash	Total	Pretreatment yield (%)
**Untreated**	35.2 ± 0.9	24.5 ± 0.6	22.2 ± 0.1	20.9 ± 4.3	102.8 ± 2.6	100.0 ± 5.1
**H_2_SO_4 _1%**	51.2 ± 0.2	7.8 ± 0.7	29.5 ± 0.6	12.2 ± 1.5	100.7 ± 1.5	93.5 ± 2.7
**NaOH 0.25%**	66.0 ± 0.5	5.2 ± 0.1	25.2 ± 0.3	3.3 ± 0.1	99.7 ± 0.9	90.5 ± 3.2
**NaOH 0.5%**	68.0 ± 1.3	3.3 ± 0.1	23.1 ± 6.7	4.3 ± 5.9	98.8 ± 0.5	84.5 ± 3.7
**NaOH 1%**	81.6 ± 0.6	3.1 ± 0.1	11.0 ± 0.9	1.9 ± 0.3	97.7 ± 1.1	72.5 ± 2.8
**NaOH 2%**	84.7 ± 0.3	3.3 ± 0.1	9.5 ± 0.5	1.1 ± 0.4	98.8 ± 1.1	68.3 ± 2.0
**NaOH 3%**	85.3 ± 0.1	3.2 ± 0.1	9.5 ± 0.5	2.3 ± 0.2	100.1 ± 0.4	70.7 ± 1.8
**NaOH 4%**	83.4 ± 3.8	3.2 ± 0.1	9.3 ± 0.4	1.8 ± 0.4	97.7 ± 4.6	65.9 ± 4.7

Contents are expressed on a bagasse dry weight basis as an average (± standard deviation) of duplicate determinations.

Untreated bagasse has 35% cellulose and similar amounts of hemicellulose (25%) and lignin (22%), as shown in Table 1. The cellulose amount increased continuously after each acid or base pretreatment, ranging from an initial 35% content to *circa *85% under pretreatments using NaOH 2% or higher. Most of the hemicellulose fraction was removed using acid, as shown by its percentage decrease from *circa *25% to 7.8% in Table 1. Smaller hemicellulose fractions were removed in the subsequent base steps, reaching minimum values for NaOH 0.5% or higher. Finally, the lignin relative percentage in the sample increased slightly with acid pretreatment due to the removal of other components (mainly hemicelluloses) and then decreased progressively with pretreatments using NaOH concentrations between 0.5% and 2%.

Ash percentage was quite high (approximately 20%) when compared to results found in the literature for other bagasse samples [35,36]. The reason for this high amount was that the samples were analyzed as received from the mill, without being washed. Therefore, they may have contained dust, soil and other debris accumulated during harvest, transportation and storage after juice extraction in the mill. These impurities were, however, removed from the sample after pretreatment steps, as shown in Table 1.

**Figure 1:**
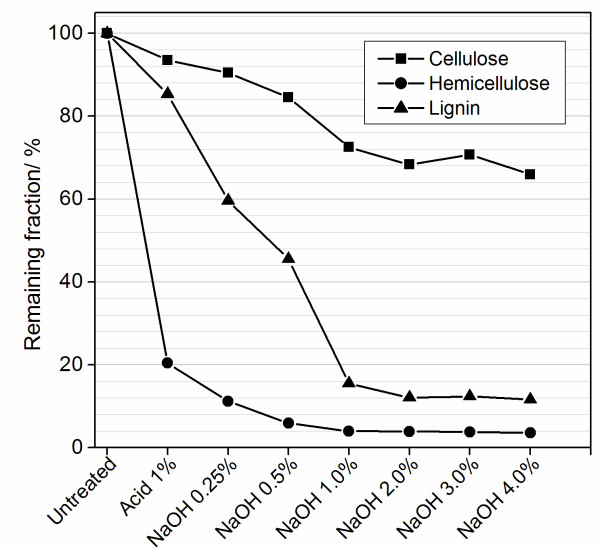
**Remaining fractions of lignocellulosic components in bagasse samples after pretreatment steps**.

In every pretreatment step, a fraction of the lignocellulosic material was removed from the solid bagasse and transferred to the hydrolysate. The remaining fraction indicates the reaction yield of each component in the sample. Figure 1 shows the fractions of cellulose, hemicellulose and lignin in the bagasse after each pretreatment. In order to calculate the remaining fractions after alkaline pretreatments, the amount of each component removed during both the acid and the base steps were taken into account.

Figure 1 shows that 80% of the hemicellulose was removed from the bagasse with the first acid pretreatment. Further hemicellulose removal was achieved using increasing NaOH concentrations, until a remaining minimum of 4% was obtained with NaOH 1%. Figure 1 also demonstrates that the first acid step reduced 15% of the total lignin of the sample. Additional lignin was gradually removed by alkaline pretreatments with increasing NaOH concentrations, reaching an 88% maximum removal using a 2% NaOH solution. Lignin extraction was therefore mostly due to the alkaline pretreatment, using NaOH concentrations between 0.5% and 1%. In spite of the cellulose enrichment observed in Table 1, increasing base concentrations also removed cellulose fractions from the sample (maximum removal around 30%), as shown in Figure 1. Cellulose loss was taken into account when hydrolysis efficiency yields were calculated.

In general, the compositional analysis shows that pretreatments using NaOH concentrations above 2% do not result in further hemicellulose and lignin removal or in cellulose enrichment of the sample. Moreover, more alkaline conditions can represent higher cellulose losses and larger process costs. NaOH concentrations above 1% might thus be unnecessary.

### Solid-state ^13^C nuclear magnetic resonance

Further information on chemical composition for bagasse samples was obtained by high resolution ^13^C solid-state NMR. The excitation of the ^13^C nuclei by a single π/2 pulse, followed by acquisition under ^1^H decoupling and fast magic angle spinning, the so called DPMAS method, is probably the most quantitative ^13^C NMR technique for organic matter applications. Nevertheless, the technique named as ramped cross-polarization under magic angle spinning and total suppression of spinning sidebands (rampCPMAS-TOSS, which we will refer to simply as CPMAS-TOSS) is more frequently used since it requires much less data acquisition time. Although the use of the radiofrequency ramp makes the magnetization transfer more uniform along the different chemical groups, the line intensities of the CPMAS-TOSS spectra are still dependent on the presence of the local ^1^H density, so they do not reflect the real amount of a given type of carbon in the samples, in short, it is not a truly quantitative method.

Despite that, CPMAS-TOSS experiments can be used for a qualitative identification of the main chemical and structural changes taking place in the samples as a consequence of the pretreatments. The use of both the total hydrolysis method for composition determination (based on HPLC and UV-visible spectroscopy measurements) and solid-state NMR is justified by their complementary features. While the first is a fully quantitative method, NMR can readily provide information on modifications taking place in specific chemical groups, without great experimental effort concerning sample preparation and analysis, which makes it faster.

**Figure 2:**
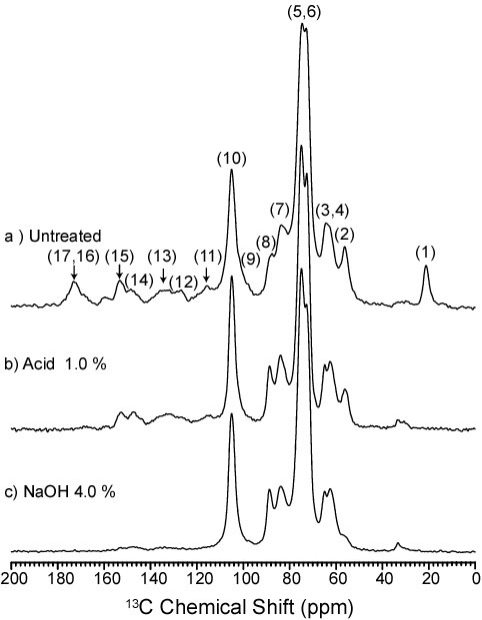
**CPMAS-TOSS NMR spectra of sugarcane bagasse**. **(a) **Untreated; **(b) **bagasse treated with acid (H_2_SO_4 _1.0%) and **(c) **bagasse treated with acid and NaOH 4.0%. The spectra were normalized by the intensity of line 10 (C1 carbon of cellulose). CPMAS-TOSS: cross polarization under magic angle spinning with total suppression of spinning sidebands; NMR: nuclear magnetic resonance.

Figure 2a shows the CPMAS-TOSS spectrum for the untreated bagasse sample. This spectrum is very similar to those obtained from wood samples [37,38], which were then used as a base for chemical shift assignments. Assignments for lines 1 to 17 (indicated in Figure 2) are presented on Table 2 and some general features are as follows:

**Table 2:** Assignments of NMR lines 1 to 17 indicated on the spectrum in Figure 2a.

Line number	Chemical group	^13^C chemical shift (ppm)
**1**	CH_3 _in acetyl groups of hemicelluloses	21.5
**2**	Aryl methoxyl carbons of lignin	56.2
**3**	C6 carbon of non-crystalline cellulose, C6 carbon of hemicelluloses, OC_γ_H_2 _carbons of lignin	62.5
**4**	C6 carbon of crystalline cellulose	64.8
**5**	C2,3,5 of cellulose, OC_α_H_2 _carbons of lignin	72.5
**6**	C2,3,5 of cellulose and hemicelluloses	74.4
**7**	C4 carbon of non-crystalline cellulose and hemicelluloses, OC_β_H_2 _carbons of lignin	83.5
**8**	C4 carbon of crystalline cellulose	87.9
**9**	Shoulder of C1 carbon of hemicelluloses	101.8
**10**	C1 carbon of cellulose	105.0
**11**	C2 and C6 aromatic carbons of syringyl and C5 and C6 aromatic carbons of guaiacyl in lignin	110-115
**12**	C2 of aromatic carbons guaiacyl in lignin	126.6
**13**	C1 and C4 aromatic carbons of syringyl (e)	134.5
**13**	C1 and C4 aromatic carbons of syringyl (ne)	136.9
**14**	C3 and C5 aromatic carbons of syringyl (ne) and C1 and C4 aromatic carbons of guaiacyl in lignin	148.0
**15**	C3 and C5 aromatic carbons of syringyl (e) in lignin	153.5
**16**	Carboxyl groups of lignin	163.0-180.0
**17**	Carboxyl groups of hemicelluloses	173.6

These assignments were based on published data [38,39] and also on the results of the spectral editing experiments described in the experimental section [40,41]. ne: nonetherified arylglycerol *β*-aryl ethers; e: etherified arylglycerol *β*-aryl ethers.

1. The most intense lines are concentrated in the 50 ppm to 120 ppm region, being attributed mostly to cellulose carbons, but also with contributions from hemicellulose and lignin signals.

2. In this spectral region, line 3 at 62.5 ppm and line 7 at 83.5 ppm are predominately due to carbons from amorphous cellulose, while the lines at 64.8 (line 4) and 87.9 ppm (line 8) are assigned to carbons in crystalline cellulose.

3. Signals from lignin are concentrated between 100 ppm to 200 ppm, and are relatively broader due to the chemically complex and disordered lignin structure.

4. The two lines at 21.5 ppm and 173.6 ppm (lines 1 and 17), are attributed to hemicellulose carbons only, while line 2 at 56.2 ppm is due to the methoxy (OCH_3_) groups in lignin structure.

Figure 2b shows the ^13^C CPMAS-TOSS spectrum acquired on a bagasse sample treated with 1% H_2_SO_4_. The absence of lines 1 and 17 (at 21.5 ppm and 173.6 ppm) is evident, confirming the almost complete removal of hemicelluloses as a result of the acid treatment. A noticeable improvement of the spectral resolution in the 50 ppm to 120 ppm region is also observed and attributed to the removal of hemicellulose lines within this region. There are no major changes in the lines assigned to lignin, in accordance with the results presented on Table 1, showing that most of the lignin contained on the bagasse remains on the sample after the acid treatment. Figure 2c shows the CPMAS-TOSS spectrum of a sample submitted to an acid followed by NaOH 4% pretreatment. A drastic reduction in the lignin lines can be noticed on this curve. Other features, such as the presence of a pronounced shoulder at 56.2 ppm and the two weak and broad lines in the 120 ppm to 200 ppm region, however, suggest that lignin still remains in the sample after the NaOH 4% pretreatment.

**Figure 3:**
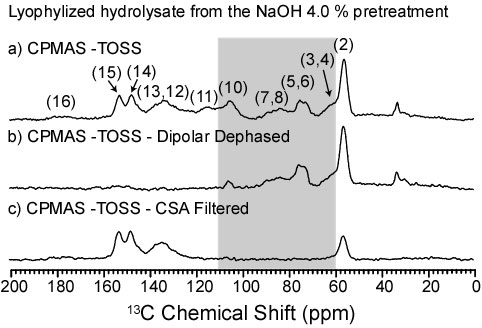
**13C NMR spectra of the lyophilized hydrolysate obtained by treating the sugarcane bagasse with acid and NaOH 4.0%.(a) **CPMAS-TOSS; **(b) **CPMAS-TOSS NMR with dipolar dephasing; **(c) **CPMAS-TOSS NMR with CSA filter. The absolute intensities are arbitrary. CPMAS-TOSS: cross polarization under magic angle spinning with total suppression of spinning sidebands; CSA: chemical shift anisotropy; NMR: nuclear magnetic resonance.

Both the solid and the hydrolysate fractions resulting from bagasse pretreatments were analyzed by NMR. Figure 3a shows the CPMAS-TOSS spectrum for the hydrolysate fraction extracted from sugarcane bagasse by the acid plus NaOH 4.0% pretreatment. The spectra in Figure 2c and 3a are thus complementary, since they refer to the remaining solid and the solubilized fraction resulting from the same pretreatment. Based on the assignments depicted in Table 2, it is possible to attribute almost all the lines in Figure 3c to lignin, which confirms that the main effect of the alkaline pretreatment is the removal of lignin from the samples. An interesting feature is that the lines in the hydrolysate spectrum keep the same relative intensity ratio as in the spectrum of Figure 2c. This shows that all the lignin components are equally extracted from the solid by the pretreatment, without preferential removal of a single component.

Despite the fact that the spectrum of Figure 3a can be almost fully assigned to lignin, the presence of particularly strong signals with chemical shifts of 72.5 ppm and 74.4 ppm indicate that part of the cellulose is also removed by the NaOH 4.0% pretreatment. To clarify that and to confirm the assignments of the lignin peaks in Table 2, NMR experiments using dipolar dephasing and chemical shift anisotropy (CSA) filters were performed. More detailed information on these experiments, as well as the pulse sequences used, can be found in references [39,40] and in the supplementary information (see Additional file 1). Simply put, in the dipolar dephasing experiment, the ^1^H dipolar decoupling is interrupted for a time period t_deph _(40 μs in this case) before signal acquisition, so that the signals from ^13^C nuclei dipolar coupled to ^1^H rapidly decay. Thus, the resulting ^13^C NMR spectrum is selective for carbons which are relatively far from ^1^H (at distances higher than approximately 3 Å) or sited in molecular segments of high mobility, which also experience reduced ^1^H dipolar coupling.

The dipolar dephased CPMAS-TOSS spectrum of the hydrolysate fraction obtained with the NaOH 4.0% pretreatment is shown in Figure 3b. As can be observed, except for the line at 56.2 ppm (which corresponds to the mobile OCH_3 _groups), most of the lignin lines are removed from the spectrum. Considering that the O-aromatic carbons are non-protonated, the decrease of these lines is mostly due to the interaction of ^13^C nuclei with intermolecular ^1^H nuclei, which shows that the lignin supra structure is heavily packed. However, the most interesting features of the spectrum shown in Figure 3b are the signals at 62.5 ppm, 64.8 ppm, 72.5 ppm, 83.5 ppm and 105 ppm (lines 3 to 5, 7 and 10, respectively - highlighted region in Figure 3). Since all the lignin lines in this spectral region are due to the protonated carbon, they do not contribute to the dipolar dephased spectrum, and the chemical shifts perfectly match cellulose peaks. This unambiguously confirms that cellulose is also removed from the sample by the NaOH 4.0% pretreatment. The CSA filtered CPMAS-TOSS spectrum of the same sample is shown in Figure 3c. In this spectrum, only those carbons with high chemical shift anisotropy appear, including carbon nuclei in aromatic, carbonyl, carboxyl and methoxyl groups. The lines attributed to cellulose and non-aromatic carbons of lignin are thus removed by the CSA filter, confirming that the assignment presented in Table 2 is correct.

**Figure 4:**
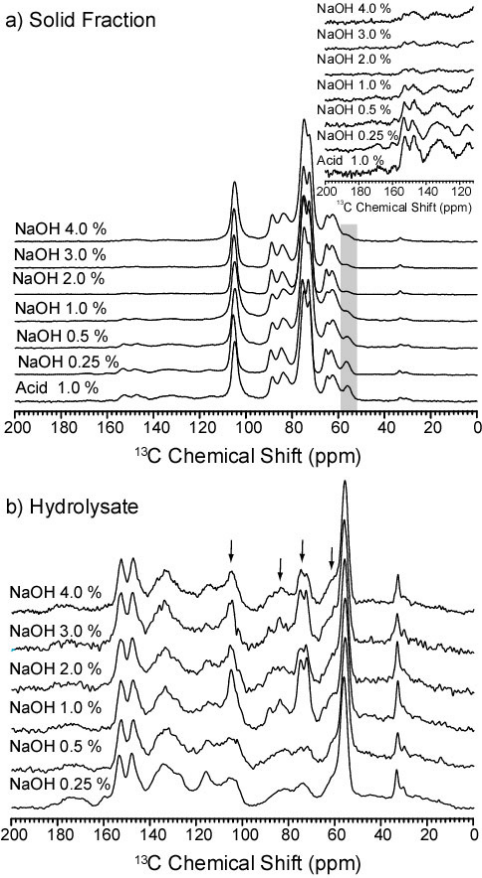
**13C CPMAS-TOSS spectra of bagasse samples treated with different NaOH concentrations**. **(a) **Remaining solid fraction and **(b) **the corresponding lyophilized hydrolysate. The inset in (a) is a zoom of the 110 ppm to 120 ppm region. Spectra in (a) were normalized by the intensity of line 10 (C1 carbon of cellulose) and in (b) by the intensity of the line at 56.2 ppm (lignin methoxy). CPMAS-TOSS: cross polarization under magic angle spinning with total suppression of spinning sidebands.

CPMAS-TOSS spectra of solid fractions remaining from bagasse samples treated with NaOH concentrations of 0.25%, 0.5%, 1.0%, 2.0%, 3.0% and 4.0%, as well as the corresponding lyophilized hydrolysates resulting from the treatments are shown in Figure 4. The solid fraction spectra (Figure 4a) exhibit a progressive decrease of the lignin lines with pretreatments using increasing NaOH concentrations (note particularly the methoxy carbon at 56.2 ppm inside the highlighted region). Besides, for NaOH concentrations higher than 2%, further reduction in the lignin amount is not detected in the NMR spectra (see the amplified region from 110 ppm to 200 ppm on the inset in Figure 4). Concerning the hydrolysate spectra in Figure 4b, the cellulose signals at 62.5 ppm, 64.8 ppm, 72.5 ppm, 83.5 ppm and 105 ppm (indicated by arrows in Figure 4b) are not observed in samples pretreated with NaOH concentrations below 0.5%, but these lines clearly show up for higher NaOH concentrations. Therefore, despite the fact that maximum lignin removal is achieved with NaOH 2%, cellulose fractions are also hydrolyzed for base concentrations above 0.5%.

### Diffuse reflectance Fourier transformed infrared spectroscopy

Diffuse reflectance Fourier transformed infrared spectroscopy (DRIFT) is another analytical technique that can be used to thoroughly understand the changes in cell wall components, particularly lignin, taking place as a consequence of different pretreatment conditions. DRIFT spectra of untreated sugarcane bagasse and samples treated with different NaOH concentrations were obtained and are presented in Additional File 2 of this paper. DRIFT data also show lignin removal from the bagasse and cellulose enrichment as the sample is treated with increasing NaOH concentrations, in accordance with the NMR and chemical characterization analyses.

### Morphological changes during pretreatment

**Figure 5:**
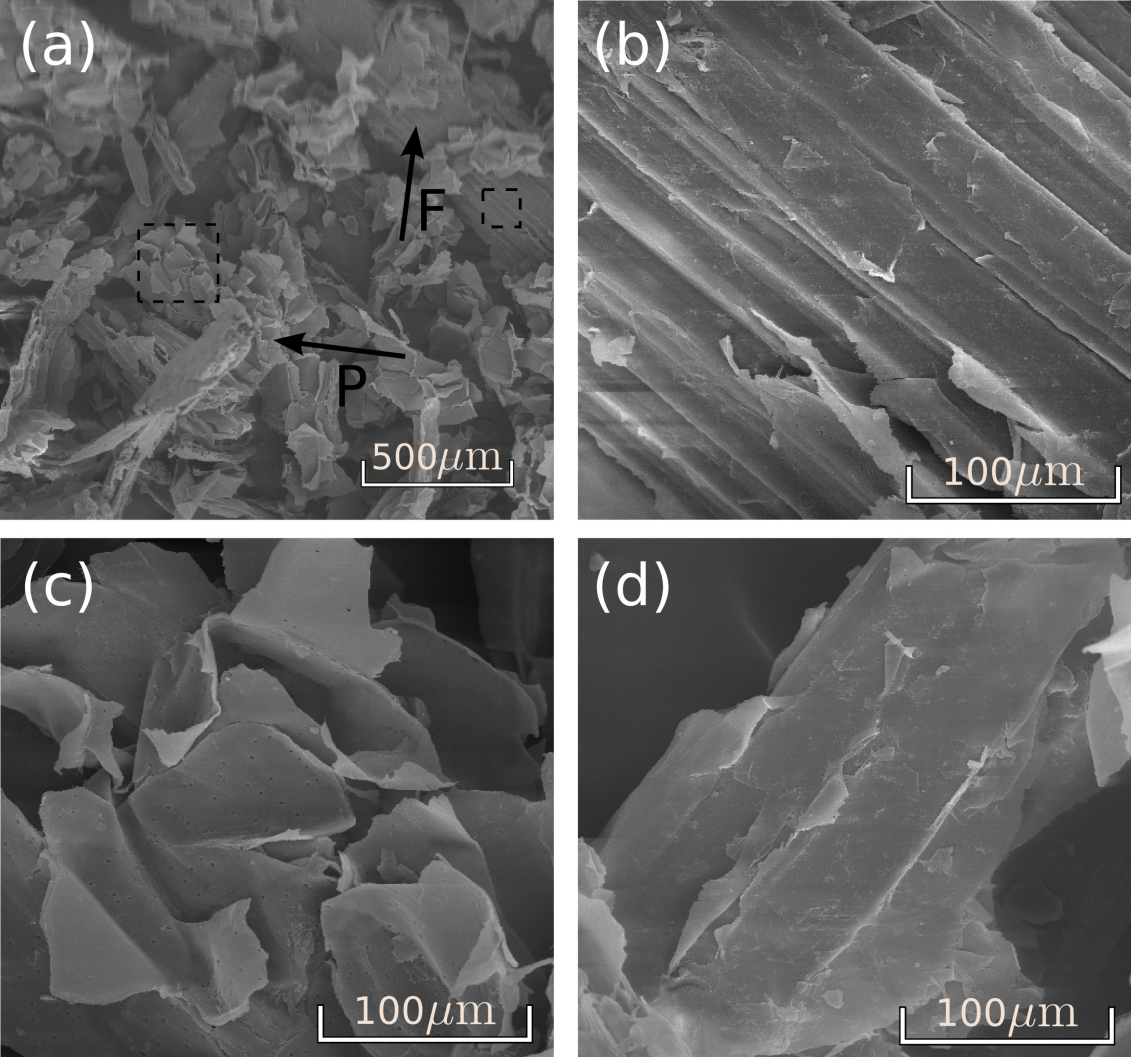
**Surface images of the untreated sugarcane bagasse obtained by scanning electron microscopy**. **(a) **General view of the sample showing fibers and pith (arrows F and P); **(b) **amplifications on the fiber surface, with parallel stripes covered by residues; **(c) **amplification on the pith; and **(d) **amplifications on the fiber surface, with parallel stripes covered by residues. Samples underwent roll and knife milling.

**Figure 6:**
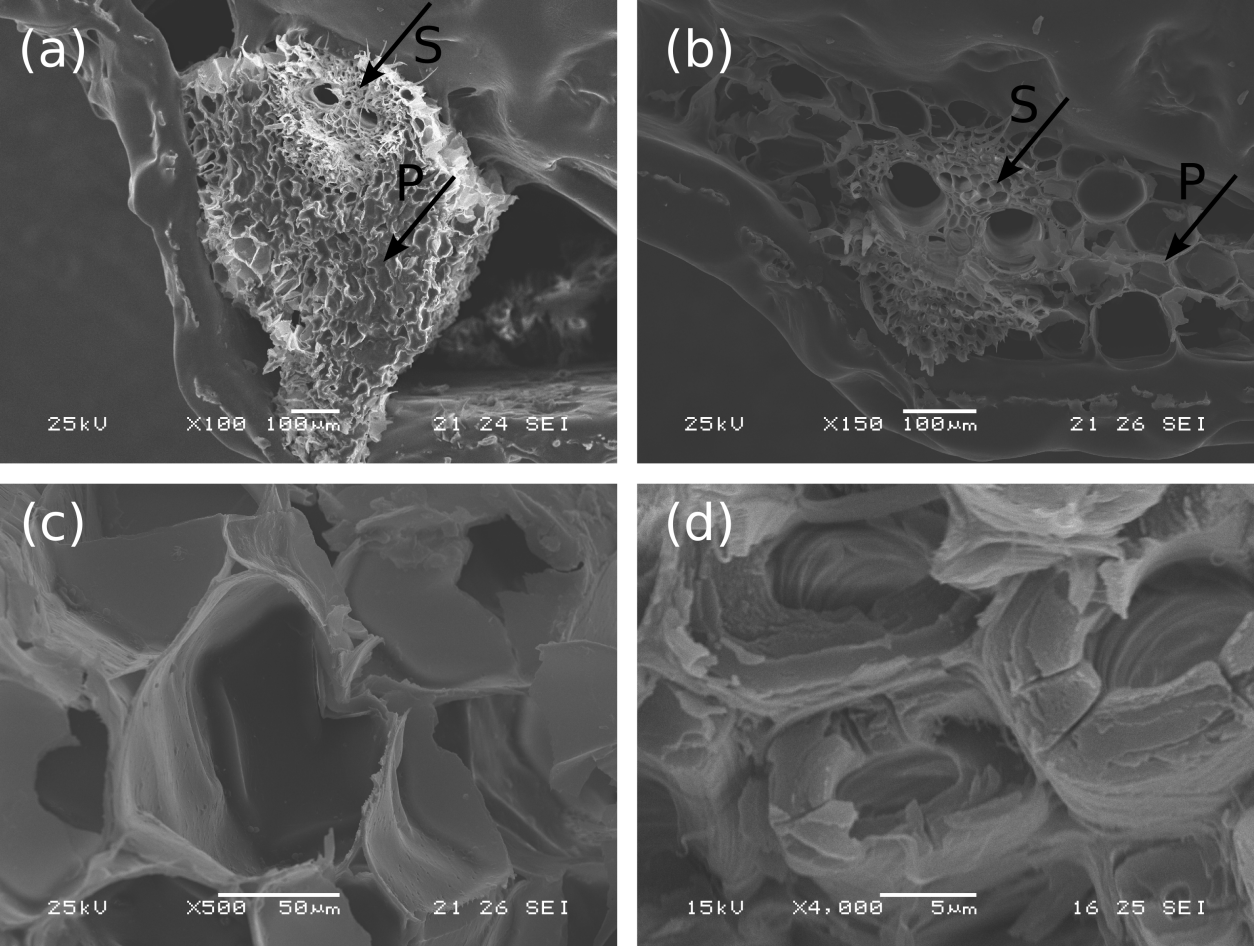
**Scanning electron microscopy images obtained from fractures of untreated sugarcane bagasse**. **(a) **General view of a bark fracture; and **(b) **fracture of the stalk core, both showing conducting vessels surrounded by sclerenchyma (arrow S) and embedded in parenchyma (arrow P); **(c) **amplification on parenchyma and **(d) **on sclerenchyma cells. Samples underwent roll milling only.

Images obtained by scanning electron microscopy on the surfaces of milled raw bagasse revealed two main morphological features: fiber structures and pith, as indicated by the arrows F and P in Figure 5a. Amplifications of the fiber and the pith regions (dashed areas in Figure 6a are shown in Figure 5b,c. The fiber surface is formed by parallel stripes and is partially covered with residual material (Figures 5b,d). In contrast, pith is a more fragile and fragmented structure containing pits, which are small pores connecting neighboring cells on the surface of the walls (Figure 5c).

Sample fractures of the bark and the sugarcane stalk cores were also obtained for samples prepared in the laboratory, starting from non-milled stalks. The bark and the internal part (core) of the stalk were separated, and then only the core was milled, using a less destructive roll mill. Sample fractures of the bark and the core of sugarcane stalk are shown in Figure 6a,b. In spite of the macroscopic differences observed between these structures, microscopically their fractures are very similar, showing vascular bundles surrounded by sclerenchyma and embedded in parenchyma. These two tissues are indicated by S and P in Figure 6a,b. In Figure 6c, parenchyma cells are amplified, showing thin walls containing pits and great internal spaces (approximately 100 μm diameter). Amplification on the sclerenchyma region (Figure 6d) shows cell walls about 5 μm thick, containing an internal lumen with diameter between 5 μm and 10 μm.

**Figure 7:**
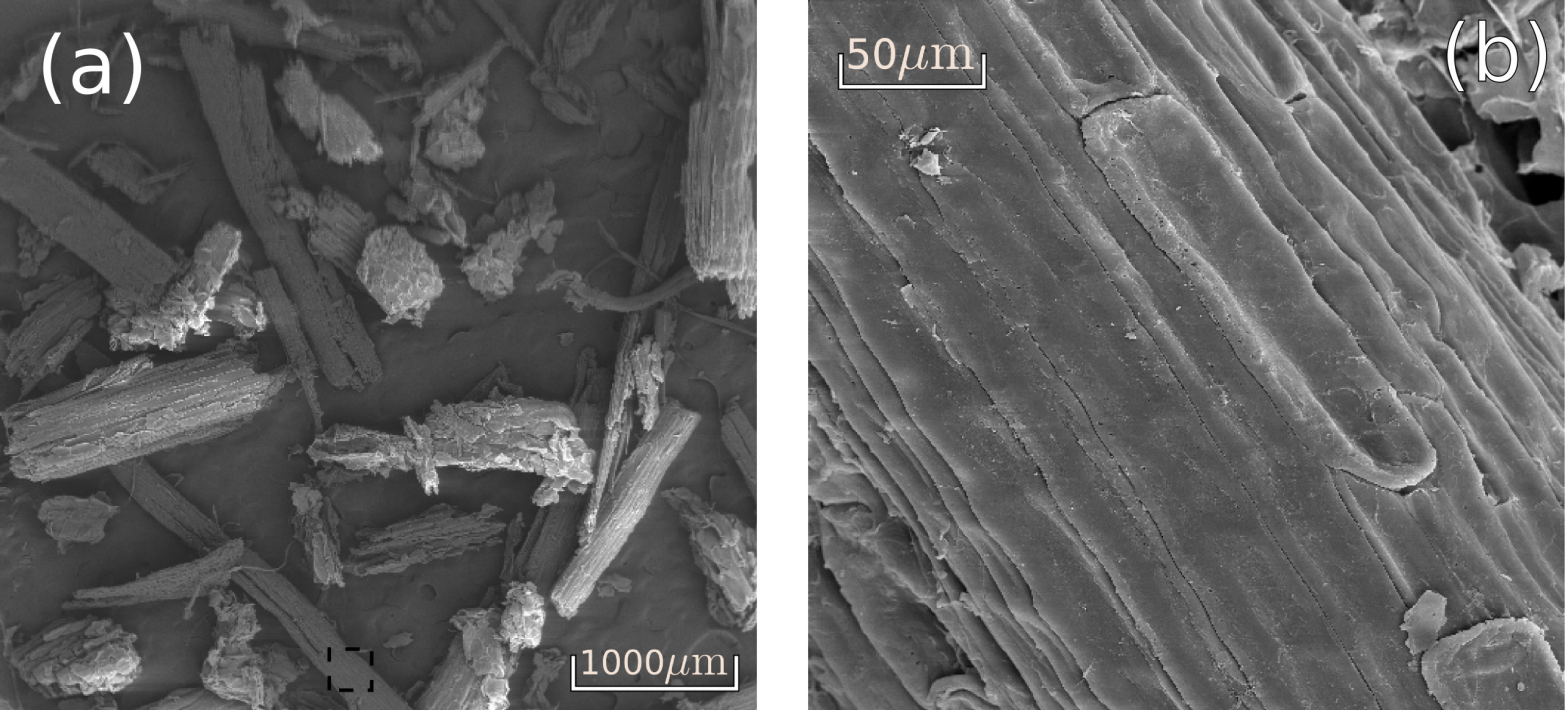
**Scanning electron microscopy surface images of the sugarcane bagasse treated with acid**. **(a) **General view of the sample showing fibers (mainly) and pith; **(b) **amplification on the fiber surface (dashed area in (a)), showing more exposed parallel stripes. Samples underwent roll and knife milling before the treatment.

After acid treatment, the morphological analysis of bagasse samples shows that the amount of pith was reduced considerably, which indicates that pith is less resistant to acid degradation than the fiber structures that prevail (Figure 7a). Fiber surfaces were also changed by the acid treatment: surface residual pith was removed and the parallel stripes appear more exposed (Figure 7b).

**Figure 8:**
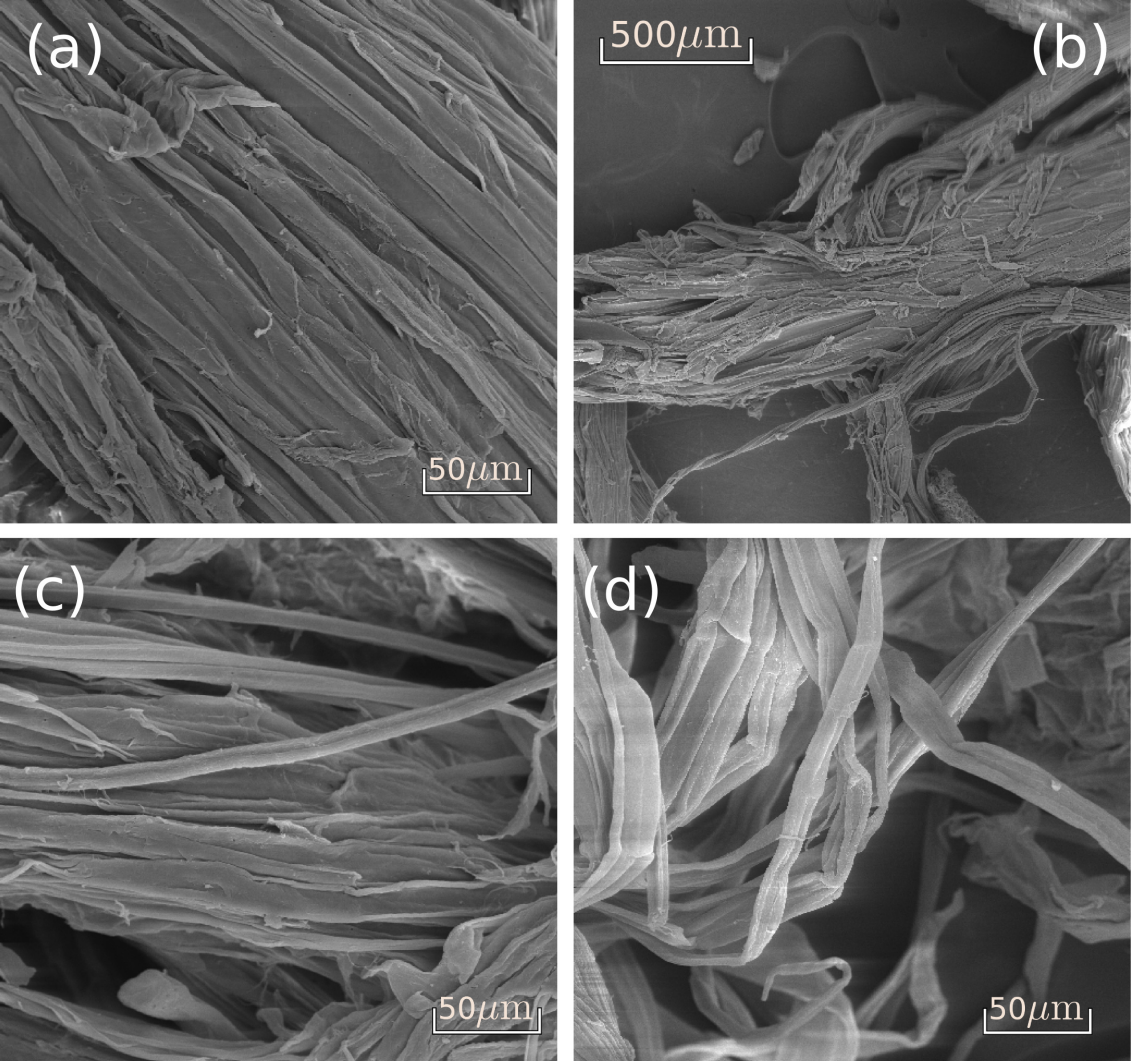
**Scanning electron microscopy surface images of the sugarcane bagasse sample treated with alkaline solutions**. **(a) **NaOH 0.5% with bundles starting to come apart; **(b) **and **(c) **NaOH 2%, showing unstructured and unattached bundles; and **(d) **NaOH 4%, showing individual fibers. Samples underwent roll and knife milling before the treatment.

Alkaline pretreatments also had a remarkable effect on the bagasse morphology, especially on the fiber bundles. Images obtained from fiber surfaces on milled samples under variable NaOH concentrations are presented in Figure 8. Using mild alkaline conditions (NaOH concentrations lower than 0.5%), bagasse bundles start to dismantle and the fibers become detached from the others (Figure 8a).

Higher NaOH contents resulted in bundles which were even more unstructured, with completely unattached and independent fibers, as shown for a sample treated with NaOH 2% in Figure 8b,c. In some regions of the sample, the bundle structure is completely lost, as shown in Figure 8d for a sample treated with NaOH 4%. Separated bundles in this sample also appeared more flexible due to their curved and twisted appearance (Figure 8d), in accordance with the observations made while this sample was being handled. Significant morphological differences were not observed between the samples treated with NaOH concentrations between 1% and 4%. Regions which were more or less affected by the treatment can be equally found in any of these samples.

The internal structure of the base treated bagasse was also obtained from fractures of samples prepared in the laboratory (such as the ones shown in Figure 6). These are more easily fractured than the knife milled samples (from Figure 8) because their initial size is relatively large and thus degradation effects are less significant. These samples are very useful for understanding the degradation process as a whole, but comparisons with the samples from Figure 8 should be made cautiously, as size reduction and mechanical stress contribute to enhanced degradation.

**Figure 9:**
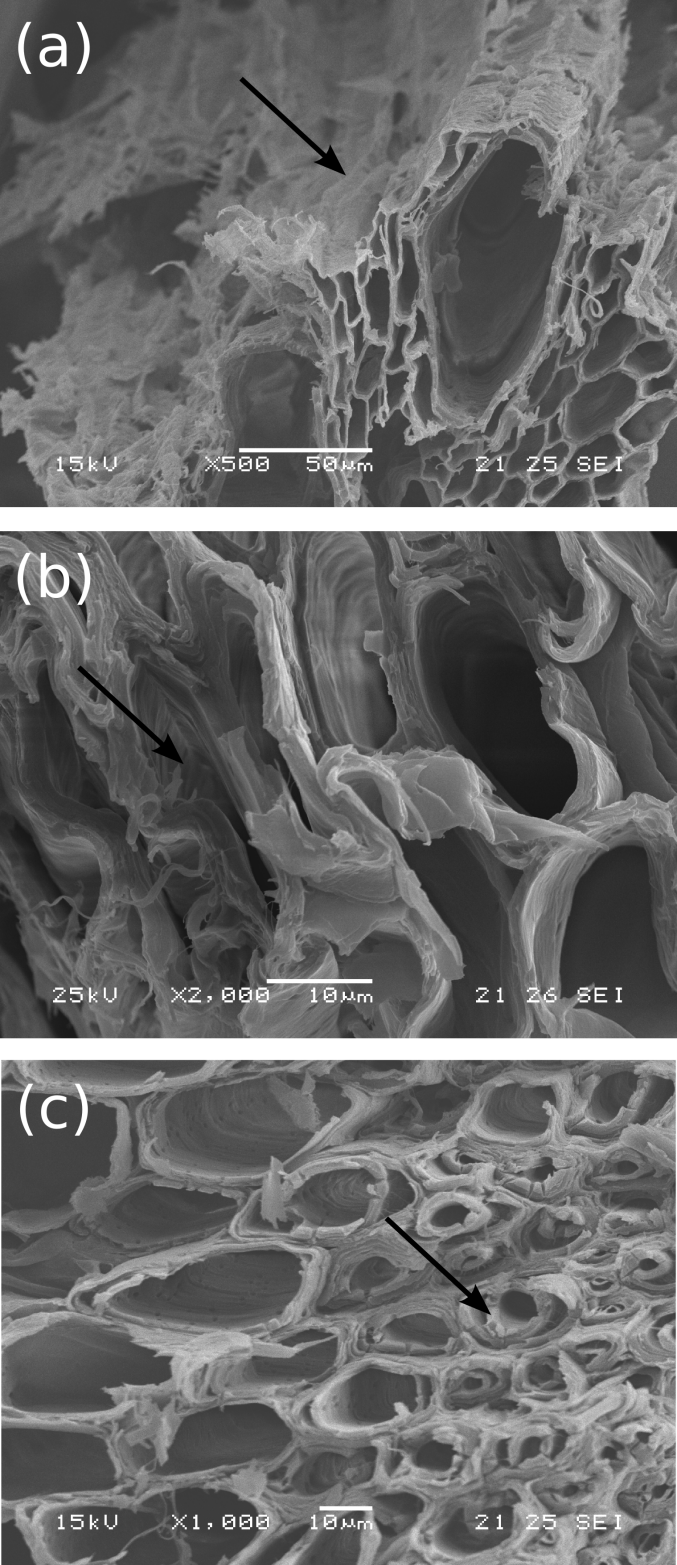
**Scanning electron microscopy fracture images of the sugarcane bagasse sample treated with alkaline solutions**. **(a) **NaOH 1%, showing the damaged surface of the cell wall; **(b) **NaOH 1%, with collapsed cell walls; **(c) **NaOH 0.5%, cell wall unstructured by peeling off radial layers. Samples underwent roll milling only.

Figure 9 shows fracture images of bagasse stalks after alkaline treatment with NaOH 0.5% and 1%. In Figure 9a, a general view of the sample is presented, and it shows a conducting vessel surrounded by cell bundles that are still joined. Tissue integrity is thus maintained to some extent, but signs of degradation are evident on the surface of the wall, as indicated by the arrow in 9a. Delignification results in hole formation in the cell wall structure and therefore its surface appears more fragile when compared to untreated samples in Figure 6c. Evidence of mechanical resistance loss in this sample can also be observed in Figure 9b, where the cell walls appear collapsed and damaged. In some regions of the samples, cell walls seem to peel off, detaching their radial layers, as pointed out by the arrow in 6c. This is probably caused by the removal of lignin fractions from the inner parts of the wall as a consequence of NaOH action.

### X-ray diffractometry

**Figure 10:**
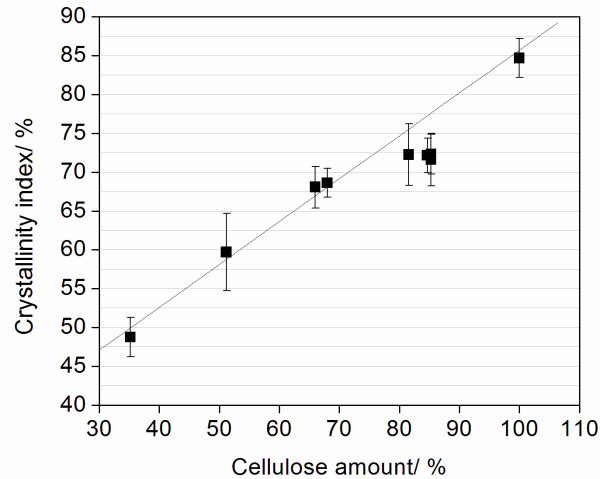
**Crystallinity index (%) calculated for each sample as a function of the cellulose amount (%)**. The error bars are standard deviations from the average values of duplicate determinations.

Sample crystallinity before and after pretreatment was determined by X-ray diffractometry measurements. The crystallinity index (CI) for all the samples was calculated according to the procedure proposed in [41,42], which considers the relative intensities of the 002 peak for cellulose I and the minimum dip between the 002 and the 101 peaks, attributed to the amorphous region. This is a fast and practical method for estimating the relative crystallinity, and is very useful for comparisons between samples. Figure 10 shows the relative CI calculated for bagasse samples as a function of their cellulose percentages. CI obtained for a commercial sample containing 100% of crystalline cellulose (Avicel - Sigma-Aldrich, St. Louis, MO, USA) is also shown for the sake of comparison. Correspondence between the cellulose amount in the sample and the pretreatment method that was applied can be found in Table 1.

The raw bagasse sample had a CI of 48.7 ± 2.5%, corresponding to a cellulose amount of 35.2%. Sample crystallinity increases linearly with the cellulose amount as the sample is treated with H_2_SO_4 _1%, NaOH 0.25% or 0.5%, corresponding to cellulose percentages of 51%, 66% and 68%, respectively (shown in Figure 10). A linear relation is also found between these values and the CI of the sample containing 100% of cellulose (Avicel, CI = 84.7 ± 2.5%). Small deviations from this linear behavior are observed in samples containing 80% to 90% of cellulose, corresponding to alkaline treatments using NaOH concentrations between 1% and 4%. A slight decrease in the crystallinity degree (3% to 5%) occurs in these samples. This result might indicate that harsher alkali pretreatments lead to a decrease in the crystallinity of the samples.

### Enzymatic hydrolysis

**Figure 11:**
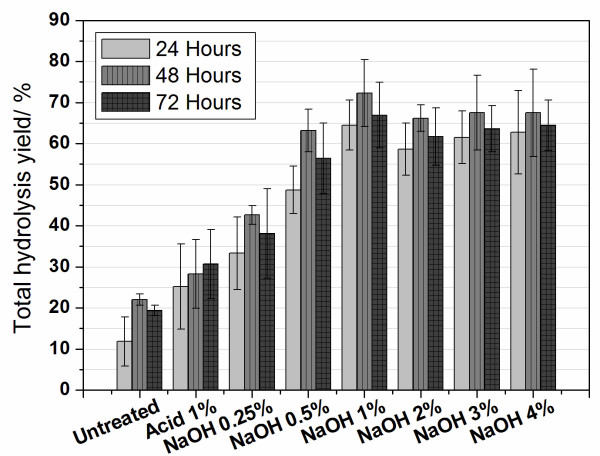
**Total cellulose conversion for untreated sugarcane bagasse and bagasse after acid and acid/alkali treatments after 24 hours, 48 hours and 72 hours hydrolysis**. The error bars are standard deviations from the average values of duplicate determinations.

The efficiency of pretreated samples to produce fermentable sugars was evaluated by measuring the total amount of glucose released from the samples after 24 hours, 48 hours and 72 hours of enzymatic hydrolysis. The hydrolysis yield (or percentage of cellulose conversion) was calculated for each step (partial hydrolysis yield) and also for the total process (total hydrolysis yield). In the first case, the total glucose liberated by a sample was divided by its cellulose amount, while in the second case, the partial hydrolysis yield was multiplied by the pretreatment yield (considering the cellulose remaining fraction of Figure 1). In this case, the total hydrolysis yield also takes into account losses of cellulose fraction occurred during pretreatments. Table 3 shows the yields obtained for partial and total hydrolysis of bagasse samples after a 48 hour reaction. Total hydrolysis yields obtained for the samples after 24 hours, 48 hours and 72 hours are shown in Figure 11.

**Table 3:** Results for partial and total hydrolysis yields obtained from bagasse samples after 48 hours under enzyme action.

Bagasse samples	Enzymatic hydrolysis (48 hours)	
	
	Partial hydrolysis yield (%)	Total hydrolysis yield (%)
**Untreated**	22.0 ± 0.3	22.0 ± 1.4

**H_2_SO_4 _1%**	30.3 ± 0.3	28.3 ± 8.4

**NaOH 0.25%**	47.1 ± 0.9	42.6 ± 2.3

**NaOH 0.5%**	79.0 ± 2.5	66.2 ± 5.2

**NaOH 1%**	99.8 ± 7.4	72.3 ± 8.1

**NaOH 2%**	96.9 ± 1.9	66.2 ± 3.3

**NaOH 3%**	95.5 ± 10.5	67.5 ± 9.1

**NaOH 4%**	97.4 ± 19.9	67.5 ± 10.6

Hydrolysis yield values are expressed as an average (± standard deviation) of duplicate determination.

Values of hydrolysis efficiency by cellulase action presented in Table 3 show that cellulose conversion is highly improved as the samples undergo the two-step pretreatment used in this work. While only 22% of the available cellulose was converted to glucose in an untreated bagasse sample (partial hydrolysis yield in Table 3), almost total cellulose conversion (95% to 100%) was achieved in samples treated with acid and NaOH concentrations above 1%. Considering cellulose losses during pretreatments, total hydrolysis yields reach maximum values between *circa *66% to 72% for NaOH concentrations of 0.5% or higher, as a result of a large increase in cellulose accessibility in these samples.

Figure 11 also shows that the differences on the total hydrolysis yield are more dependent on the different pretreatment conditions than on the hydrolysis time, considering the experimental conditions between 24 hours and 72 hours tested here. This can be explained by the high amount of enzymes used in these experiments that already results in high hydrolysis yields after only 24 hours of incubation.

According to the values for total cellulose conversion presented in Table 3 and Figure 11, acid treatment followed by the step with NaOH 1% seems to be the best pretreatment condition to be used on bagasse samples prior to cellulase action within the range of conditions studied in our experiments. Hydrolysis yield values are improved with the treatments until this condition and higher NaOH concentrations do not result in further improvement in cellulose conversion.

## Discussion

In line with previous studies [43], our morphological analysis of sugarcane bagasse shows that it is formed by a complex structure containing vascular bundles (for xylem and phloem), reinforced by sclerenchymatous cells and embedded in parenchyma (Figure 6). During the industrial process for ethanol and sugar production, bagasse samples are severely fragmented as a result of knife and roll milling and, therefore, only some parts of the total structure can be recognized in the images obtained on post-processed samples. The main features observed in the milled bagasse are fibers and pith, as shown in Figure 5. Fiber structures are formed by vascular bundles surrounded by sclerenchyma, which is a lignified tissue [44], relatively resistant to acid action. On the other hand, pith residues observed in Figure 5a and also the residues on the fiber surfaces (Figure 5b,d) are less resistant to the acid treatment and must be mainly formed by soft walls from parenchyma cells, knife milled and fragmented during sugarcane industrial processing. This notion is reinforced by the images containing pits given in Figure 5c. As a consequence of lignin and hemicellulose removal in the acid pretreatment, cellulose fraction of the samples increases from 35% to 51%.

After hemicellulose and partial lignin extraction in the acid step, alkaline treatments using increasing NaOH concentrations are then performed with the aim of removing further lignin from the sample. Alkaline pretreatments proved to be very efficient: up to 85% lignin fractions are removed from the solid fraction using solutions with a 1% NaOH concentration, as displayed in Figure 1. Lignin removal is confirmed by solid-state ^13^C NMR experiments, which show lignin signals decreasing in the solid bagasse treated with increasing NaOH concentrations, together with lignin lines showing up on the spectra of the hydrolysate resulting from the treatment (Figure 4). Lignin removal is also clear from DRIFT measurements, which show a decrease in the intensity of the lignin main peak at 1510 cm^-1 ^(see Figure 1A in Additional file 2).

Hemicellulose fractions are also removed by alkaline treatments, reaching a maximum removal limit of approximately 96% using NaOH concentrations equal to 1% or higher. As a rule, considering the relative percentages of the three lignocellulosic components, pretreatment conditions using NaOH concentrations lower than 1% seem to be more efficient for the purposes of this work. NaOH concentrations higher than 1% do not result in further removal of any of the lignocellulosic components, and their use is thus unnecessary and uneconomic. Specifically, the pretreatment using NaOH 1% results in maximum removal of hemicellulose and lignin, considering the experimental conditions used in this work.

On the other hand, a maximum of approximately 30% of cellulose is also lost under the alkaline conditions used here, as revealed by HPLC and NMR measurements. This can be understood as a consequence of the tangled configuration involving the lignocellulosic components. Removing the restraining components from the lignocellulosic matrix without losing cellulose depends on the chemical and spatial relationship among the components and will certainly influence the final hydrolysis yields.

Sun and collaborators obtained lignin and hemicellulose removal from wheat straw, with minimum cellulose loss, using an alkaline treatment. These authors tested the effect of reaction temperature, time, alkali concentration and type, and achieved a maximum of 82.6% and 59.2% of hemicellulose and lignin losses, respectively, using NaOH 1.5%, at 20°C for 144 hours [22,25]. Almost no cellulose was lost under these experimental conditions, but the total hemicellulose and lignin amounts were also lower than the ones removed here, using NaOH 1% at 120°C for 1 hour (96% and 88%, respectively).

Comparable results to those obtained in the present work were reported by Rocha and co-workers [32], who attained 90% of hemicellulose removal from dry sugarcane bagasse, with a 15% loss of the cellulose contained in the sample, using a pretreatment method based on a mixture of sulfuric and acetic acids. This would be the equivalent of using H_2_SO_4 _1% followed by NaOH 0.5% in the two-step method applied here, resulting in 94% of hemicellulose removal and a 16% cellulose loss. These authors obtained a hydrolysis yield around 75%, using an enzyme cocktail containing Celluclast and Novozyme 188, a result comparable to our hydrolysis yield of 79% using Accelerase 1500 and Novozyme 188.

The advantage in applying the two-step treatment proposed in this work is that a great amount of lignin can also be removed from the sample (85%), compared to a maximum lignin removal of 5% for the treatment using acids only [32]. This efficient lignin removal allows the conversion of almost all the cellulose contained on the sample (95% to 100%).

In terms of morphological changes, lignin removal from bagasse samples resulted in the separation of cell bundles, forming long cellular structures which are well connected in the longitudinal direction, but separated from neighboring bundles, as shown in Figure 8. Cell bundles that were tightly packed together before the base treatments (Figures 5, 6 and 7), started to dismantle under NaOH concentrations lower than 0.5%, becoming totally separated in some regions of the sample under higher NaOH concentrations. These results show that lignin is the substance gluing the sugarcane bundles together, in accordance to what was also proposed by Fromm *et al*. for spruce and beech wood cells [45]. These authors used transmission electron microscopy and potassium permanganate to dye lignin-rich regions in wood samples and observed that the highest lignin levels were concentrated in the middle lamella (50% to 100%), the membrane binding neighbouring cells. According to these authors, the secondary wall has a much lower lignin concentration of approximately 20% to 25% [45].

In addition, the gap between cellulose microfibrils inside the walls is filled with a thin layer of the lignin-hemicellulose complex [46,47]. Removing lignin from the inner parts of the cell wall consequently resulted in a damaged and porous morphology, as observed in Figure 9, followed by a loss of mechanical resistance (Figures 8d and 9). These results show that lignin removal lead to the unstructuring of the sugarcane cell wall through a process that occurs in two levels. The first level refers to the loss of cohesion between neighbouring cell walls, while the second one corresponds to degradation inside the wall itself, caused by peeling off and hole formation.

All these morphological changes are important for improving enzymatic hydrolysis efficiency, as shown in Table 3. Enzymatic action is hindered when the bagasse fibers are packed and their surfaces are protected as in Figures 5b,d and 6, resulting in a hydrolysis efficiency of only 22% after 48 hours of hydrolysis. Hemicellulose removal and the cleaning of the fiber surfaces (Figure 7b) resulted in an increased cellulose conversion of about 30%. However, the most efficient results for cellulose conversion appear when the morphological changes cited above take place. At a concentration of 0.5% NaOH, when the bundle dismantles and peels off, the conversion rate increases to 79%, and as these two processes advance (Figures 8b,c,d) and 9), cellulose conversion is close to the totality (95% to 100%) after 48 hours of enzyme action.

Even considering the unavoidable cellulose loss that occurs during pretreatment, the hydrolysis yield is remarkably improved, passing from 22% in the raw bagasse to 72% as the sample is treated with H_2_SO_4 _then NaOH 1%.

Apart from morphological changes, other factors may also be contributing to improve cellulase action as the lignin fraction is removed from the bagasse samples. Lignin may hinder cellulose hydrolysis by inhibiting their function or by acting as an 'enzymatic trap', which leads to an unproductive adsorption of the cellulases. This may happen not only at middle lamellae but also within the cell walls. Besides, the lower mechanical resistance may also contribute to its enhanced digestibility.

Sample crystallinity is pointed out by some authors as an important factor influencing hydrolysis efficiency [43,48]. The relative CI determined for bagasse samples increases linearly with their cellulose percentage for treatments using NaOH concentrations lower than 0.5% (Figure 10). It shows that the cellulose crystalline structure remains unchanged as the other components are hydrolyzed on these treatments. A decrease in CI values could facilitate cellulose conversion into glucose, and thus, the small deviation from linearity in Figure 10 would contribute to the increased hydrolysis efficiency observed between samples treated with NaOH 0.5% and NaOH 1% (Table 3 and Figure 11). On the other hand, it is clear from our results that cellulose crystallinity is not a dominant factor that determines biomass recalcitrance. In fact, while increase in crystallinity of the samples correlates with the increase of NaOH concentration during the second pretreatment step, the enzymatic hydrolysis yields reach their maximum at NaOH 1% pretreatment and stay at this level further on. If crystallinity is a main factor in biomass recalcitrance, one would expect to see a negative correlation between biomass crystallinity and enzymatic hydrolysis yields, which is clearly not the case. We cannot exclude, however, that the kinetics of the biomass hydrolysis at shorter times (less than 24 hours) could be affected by the biomass crystallinity or that other factors, such as an increased surface area, may be compensating for the increase in CI.

## Conclusions

The two-step pretreatment used in this work removed up to 96% and 85% of hemicelluloses and lignin from sugarcane bagasse sample, and was thus very successful in producing a cellulose-rich matrix for enzymatic depolymerisation. The total cellulose conversion yield was 72% on samples treated with H_2_SO_4 _and NaOH 1%, representing a considerable increase in comparison to 22% for the untreated bagasse. The different physical-chemical techniques used in this work provided comprehensive information on the mechanism involved in this pretreatment sequence. The improvement in cellulose conversion is mainly due to lignin removal from the sample, which has two important effects on cell wall unstructuring: the loss of cohesion between adjacent cell walls that were initially joined by lignin and the damages inside the wall itself, such as porous and void formation, which expose the cellulose-rich areas to enzyme action.

## Methods

### Materials

Grounded sugarcane bagasse was kindly provided by the Cosan Group (Ibaté, São Paulo, Brazil) and used as received, without washing or further milling. All the experiments and measurements described in this work were performed in the same batch of samples. During the industrial process for juice extraction, sugarcane stalks underwent knife and roll milling and the resulting bagasse had variable particle sizes. Prior to pretreatments, samples were passed through a 9.8 mm sieve. Bagasse was dried in a convection oven at 60°C for 24 hours and then stored in plastic containers at room temperature and humidity until used. Sulfuric acid and sodium hydroxide for sample pretreatments were purchased from JTBaker (Mexico City, Mexico) and from Mallinckrodt Chemicals (Linköping, Sweden), respectively, and were used as received.

### Bagasse pretreatments

Sugarcane bagasse was initially hydrolyzed with diluted H_2_SO_4 _(1% v/v in water) for 40 minutes at 120°C. The pressure was kept at 1.05 bar and a 1:10 solid to liquid ratio (grams of bagasse/mL of solution) was used. This set of conditions was previously optimized for hemicellulose removal from sugar cane bagasse by Fogel *et al*. [30]. The bagasse solid fraction was separated from the hydrolysate by filtration and abundantly washed with tap water to eliminate acid excess before oven drying at 60°C for 24 hours. A second pretreatment step for bagasse delignification followed, using NaOH solutions with increasing concentrations (0.25%, 0.5%, 1.0%, 2.0%, 3.0% or 4.0% w/v), at 120°C for 40 minutes. Six pretreated bagasse samples were then obtained by filtration, thorough washing until a neutral pH was reached and solid drying in an oven for 24 more hours at 60°C.

### Chemical composition

Summative mass closure of the biomass was obtained following the protocol established by Rocha *et al*. [32], developed specifically for sugarcane bagasse, and based on standard protocols from the National Renewable Energy Laboratory [49,50] and also on a validated methodology from [51,52]. Samples were milled in a knife mill and passed through a 2 mm sieve and their humidity content was determined using a Shimadzu (Kyoto, Japan) heating balance. All the component contents are expressed on a bagasse dry weight basis as an average (± standard deviation) of duplicate determinations for each sample.

Raw bagasse was washed in 95% ethanol for 6 hours to remove extractives, using a Soxhlet tube and bags of semi-quantitative paper containing the samples. Milled samples (2 g) were treated with 10 mL of a 72% H_2_SO_4 _solution, at 45 ± 0.5°C for 7 minutes, under vigorous stirring. Then, distilled water was added to the slurry until reaching a 275 mL final volume, and the material was kept at 120°C and 1.05 bar for 30 minutes. After cooling to room temperature, it was filtered using quantitative filter papers to separate the hydrolysate from the solid fraction. The solid fraction was rinsed until a neutral pH was attained and then oven dried at 105°C to a constant weight (weight A), which corresponded to the summation of insoluble lignin and ash. Finally, filter papers containing samples were transferred to porcelain crucibles and calcinated in a muffle at 800°C for 2 hours. After cooling to room temperature inside a desiccator, the ash weight was determined (weight B), as well as the insoluble lignin amount by subtraction from A.

The soluble lignin was determined by absorbance measurements (280 nm) using a UV-VIS Perkin Elmer (Waltham, MA, USA) spectrophotometer (model Lambda 25). Calibration curves were performed with purified lignin from sugarcane bagasse and the final values for soluble lignin took into account the interfering absorption due to furfural and hydroxymethylfurfural, as previously described [32].

The hydrolysate was also analyzed by HPLC to determine carbohydrate, organic acid, furfural and hydroxymethylfurfural content. HPLC determinations were performed in a Shimadzu LC-10AD chromatograph equipped with refractive index and UV-VIS detectors. An Aminex column (HPX-87H, 300.0 × 7.8 mm, Bio-Rad - Hercules, CA, USA) was used for carbohydrate and organic acid analysis using diluted H_2_SO_4 _as a mobile phase (5.10^-3 ^mol/L, flow rate = 0.6 mL/min and temperature 45°C). Prior to injection, samples were filtered for lignin removal using a Sep-Pak C18 filter (Waters - Milfords, MA, USA).

Furfural and hydroxymethylfurfural were quantified in the same equipment using a LiChrospher column (100 RP-18, 125.0 × 4.0 mm, Hewlett- Packard - Palo Alto, CA, USA) operating with a mobile phase containing acetonitrile to water ratio of 1:8 (v/v) with 1% acetic acid at a 0.8 mL/min flow rate and 25°C. Detection was performed at a 276 nm wavelength using a UV-VIS detector (Shimadzu SPD-10).

### Solid-state NMR

Solid-state ^13^C NMR experiments were carried out in a Varian Inova spectrometer (Varian, Palo Alto, CA, USA) at ^13^C and ^1^H frequencies of 100.5 MHzand 400.0 MHz, respectively. A Jackobsen 7-mm magic-angle spinning double-resonance probe head was used. An excitation method based on ^1^H-^13^C cross-polarization with a radio-frequency ramp, denoted here simply as CPMAS, was used to achieve a good signal to noise ratio, combined with a uniform excitation among the different chemical groups [53]. Spinning sidebands were avoided using TOSS scheme with composite pulses, as described in the literature [40]. All the experiments were carried out at spinning frequencies of 5 kHz, using typical cross-polarization times of 1 ms, acquisition times of 20 ms, and recycle delays of 4 s. For identification of aromatic groups, CSA filter experiments were also performed following the procedure suggested by Mao and Schmidt-Rohr [41], using a five-pulse CSA dephasing filter, and four-pulse TOSS, but also using composite π pulses for offset compensation [40]. Dipolar dephasing experiments for identification of non-protonated carbons were carried out with a 40 μs dipolar dephasing time for CSA experiments. High power ^1^H two-pulse phase modulation decoupling [54] of 80 kHz was applied in all measurements. More information on the NMR methods is provided in Additional file 1.

NMR experiments were performed on the untreated bagasse sample and also on the solid and the solubilized bagasse fractions resulting from pretreatments. The solubilized fraction (hydrolysate) was prepared for analysis by neutralization and dialysis, followed by lyophilization.

### Scanning electron microscopy

Bagasse morphology was also analyzed by scanning electron microscopy before and after undergoing pretreatments. Samples from surfaces or transversal sections (obtained by fracture in liquid N_2_) were dried and coated with gold in a SCD 050 sputter coater (Oerlikon-Balzers, Balzers, Lichtenstein). Sample imaging was carried out using scanning electron microscopes, models DSM 960 (Zeiss, Oberkochen, Germany) or JSM 5900LV (Jeol, Akishima, Japan). A large number of images was obtained on different areas of the samples (at least 20 images per sample) to guarantee the reproducibility of the results.

Two different groups of samples were imaged: the industrial samples submitted to roll and knife milling during the juice extraction process; and the samples prepared in the laboratory by separation of the more external (bark) and the internal parts of the stalks followed by roll milling only. This second group contained larger bagasse pieces, which facilitated the identification of image features that appear in the first group. Besides, knife milled samples lose their mechanical resistance during pretreatments and are difficult to fracture. Thicker samples are thus more suitable for transversal section imaging.

### X-ray diffraction

X-ray diffraction data were obtained in a Rigaku Rotaflex diffractometer model RU200B (Tokyo, Japan) using monochromatic CuKα radiation (1.54 Å). The goniometer scanned a 2θ range between 5° and 65° at a 2°/min scanning rate. Samples were knife milled prior to analysis until they were put through a 2 mm sieve. The CI for all the samples was calculated according to the procedure proposed in [42,43]. CI was obtained from the relationship between the intensity of the 002 peak for cellulose I (I_002_) and the minimum dip (I_am_) between the 002 and the 101 peaks according to Equation 1:

(1) $$CI=\frac{I_{002}-I_{am}}{I_{002}}\times 100$$

The background subtraction was carried out considering the spectrum obtained for an empty sample holder in the same 2θ range. The CI was also calculated from a commercial sample containing 100% of microcrystalline cellulose (Avicel PH-101, Sigma-Aldrich, St. Luis, MO, USA for comparison. Samples were measured in duplicates and the average values are presented with their respective standard deviations.

### Enzymatic hydrolysis

Enzymatic hydrolysis of pretreated bagasse samples was carried out at a substrate ratio of 2.5% (w/V) in 50 mM sodium citrate buffer (pH 5.0), under a 200 rpm constant stirring and at 50°C. The enzymatic blend applied consisted of 25 filter paper units of Accellerase 1500 (Genencor, Rochester, NY, USA) and 50 Beta-Glucanase Units of Novozyme 188 (Novozymes, Bagsvaerd, Denmark) per gram of biomass. The amount of released glucose was measured by an ion chromatograph system (DX-500, Dionex, Sunnyvale, CA, USA), equipped with pulsed amperometric detection and a CarboPac™ PA1 anion exchange column. A 10 mM NaOH solution was used as the mobile phase (flow rate 1 mL/min) and glucose and cellobiose were used as standards. Glucose values were quantified in duplicate and the conversion values are expressed as an average (± standard deviation).

The cellulose hydrolysis yield (HY) was determined as described by Maeda *et al*. [35], considering the amount of released glucose (RG) in g/L and the cellulose percentage (C) in the bagasse sample, according to Equation 2:

(2) $$HY=\frac{RG\left(g∕L\right)}{\text{25}\times \text{C(\% )}\times \text{1}\text{.1}}\times 100$$

where the number 25 refers to the biomass concentration (g/L) and 1.1 is a correction factor due to the addition of water molecules to the anhydroglucose residues in cellulose.

## List of abbreviations

CI: crystallinity index; CPMAS-TOSS: cross polarization under magic angle spinning with total suppression of spinning sidebands; CSA: chemical shift anisotropy; DRIFT: diffuse reflectance Fourier transformed infrared spectroscopy; HPLC: high proficiency liquid chromatography; NMR: nuclear magnetic resonance.

## Competing interests

The authors declare that they have no competing interests.

## Authors' contributions

CAR and MAL planned and carried out the biomass pretreatments and the determination of chemical composition, as well as the analysis of the results and manuscript writing. CAR was also responsible for X-ray diffraction measurements and scanning electron microscopy sample preparation, imaging and analysis. The hydrolysis experiments and result analysis were carried out by MAL. ERA performed the NMR experiments and analysis, and contributed to the manuscript draft. DRIFT measurements and analysis were performed by both MAL and WG. PM participated in the determination of sample chemical composition. IP coordinated the overall study, and contributed to the analysis of the results and finalizing the paper. All authors suggested modifications to the draft and approved the final manuscript.

## Supplementary Material

Additional file 1 **Description of solid-state nuclear magnetic resonance methods**. This file contains detailed information on the NMR experimental approaches used in this work (pdf). Click here for file

Additional file 2 **DRIFT experiments**. This file contains results obtained on sugarcane bagasse samples using diffuse reflectance Fourier transformed infrared (DRIFT) spectroscopy (pdf). Click here for file
