# Supplementary material for: Chemical and morphological characterization of sugarcane bagasse submitted to a delignification process for enhanced enzymatic digestibility
Source: Biotechnol Biofuels. 2011 Nov 28;4:54. doi: 10.1186/1754-6834-4-54 (PMC3377919; doi:10.1186/1754-6834-4-54)
Supplement: Additional file 1 — Description of solid-state nuclear magnetic resonance methods. This file contains detailed information on the NMR experimental approaches used in this work (pdf). [file 1754-6834-4-54-S1.PDF]

Supplementary information of the paper:

Chemical and morphological characterization of sugarcane bagasse submitted to delignification process for enhanced enzymatic digestibility

Authors: Camila Alves Rezende; Marisa Aparecida de Lima; Priscila Maziero, Eduardo Ribeiro deAzevedo; Wanius Garcia; Igor Polikarpov.

Additional information 1: Description of Solid-State Nuclear Magnetic Resonance methods

In this work, high resolution ^{13}C solid-state NMR was used to obtain information on the chemical modifications taking place on sugarcane bagasse. The method consists of monitoring the change in the ^{13}C NMR spectra, which is characterized by a series of lines associated to the different chemical groups in the sample. The DPMAS method is probably the most quantitative ^{13}C NMR technique for applications in organic matter. In this method, the excitation of the ^{13}C nuclei by a single $\pi/2$ pulse is followed by the acquisition under ^{1}H decoupling (a time proportional pulse modulation scheme –TPPM was used in this case) and fast Magic Angle Spinning (MAS). However, due to the low abundance and typical long relaxation times of the ^{13}C nuclei in solid samples, an excitation scheme based on the polarization transfer between ^{1}H and ^{13}C (CP) is usually used. This procedure has the advantage of speeding up the signal acquisition 10 to 50 times, but the signal intensities become dependent on the proton density around the ^{13}C nuclei, i.e., the line intensities do not reflect the exact amount of a given type of carbon associated to that line. This problem is minimized by the use of ramped-CP that allows a more uniform magnetization transfer from ^{1}H to ^{13}C nuclei [1]. Besides this, for spinning frequencies lower than 15 kHz, spinning sidebands of chemical groups with

high chemical shift anisotropy (typical of aromatic and carboxyl groups) can overlap the lines from other groups. As a consequence, it is usually necessary to acquire NMR spectra using high spinning frequencies (>15 kHz) or, alternatively, acquire the spectrum using Total Suppression of Spinning Sidebands (TOSS) [2]. To minimize relaxation and pulse imperfection effects on the carbons, which can affect the TOSS spectra, composite π pulses were applied in the TOSS pulse sequence [3]. The influence of relaxation effects might be neglected in comparative studies. Figure A1(a) shows the pulse sequence used for the ramped CPMAS-TOSS experiments, with the individual steps rampCP and TOSS indicated.

In order to identify the ^{13}C signals corresponding to non-protonated carbons, spectral edition based on dipolar dephased ramped CPMAS-TOSS experiments [4,5] was performed. The dipolar decoupling is interrupted for a time period t_{deph} (=40 μs in this case), so that the magnetization from ^{13}C nuclei coupled to ^{1}H quickly dephase, while the magnetization from the ^{1}H non-coupled to ^{13}C remains. Thus, the resulting ^{13}C NMR spectrum is selective for carbons which are not directly coupled to ^{1}H or which are sited in molecular segments with high mobility [4]. Figure A1(b) shows the pulse sequence used in these experiments. As it can be noticed, the only difference to the ramped CPMAS-TOSS sequence is the decoupling removal during the last delay of the TOSS sequence.

Chemical Shift Anisotropy (CSA) filtered spectrum was also used to identify the different components in sugarcane bagasse samples. Here, only the carbons with high chemical shift anisotropy appear in the spectrum, including carbon nuclei in aromatic, carbonyl, carboxyl and methoxyl groups. Since the use of the MAS averages out the anisotropic part of the chemical shift interaction, the NMR frequency becomes exclusively dependent on the isotropic chemical shifts. This provides, together with the

^1H dipolar decoupling, the high resolution of the ^{13}C spectrum. However, the chemical groups can also be differentiated by their chemical shift anisotropy (CSA). Obtaining a spectrum exclusively from segments with small CSAs may be thus interesting to identify specific components of a chemically complex sample. This can be achieved by the pulse sequence shown in Figure A1(c), where a composite π pulse train (usually known as recoupling pulse) is applied right after the CP excitation. The application of the recoupling π pulse train reintroduces the evolution under the anisotropic part of the chemical shift interaction, making the ^{13}C nuclei from highly anisotropic sites (carbonyl, aromatic, etc.) dephase very fast [4]. Thus, the signal obtained after the application of these pulses arises only from carbons with small CSA. To obtain the high resolution ^{13}C spectrum, a TOSS is applied after the CSA filter as depicted in Figure A1(c).

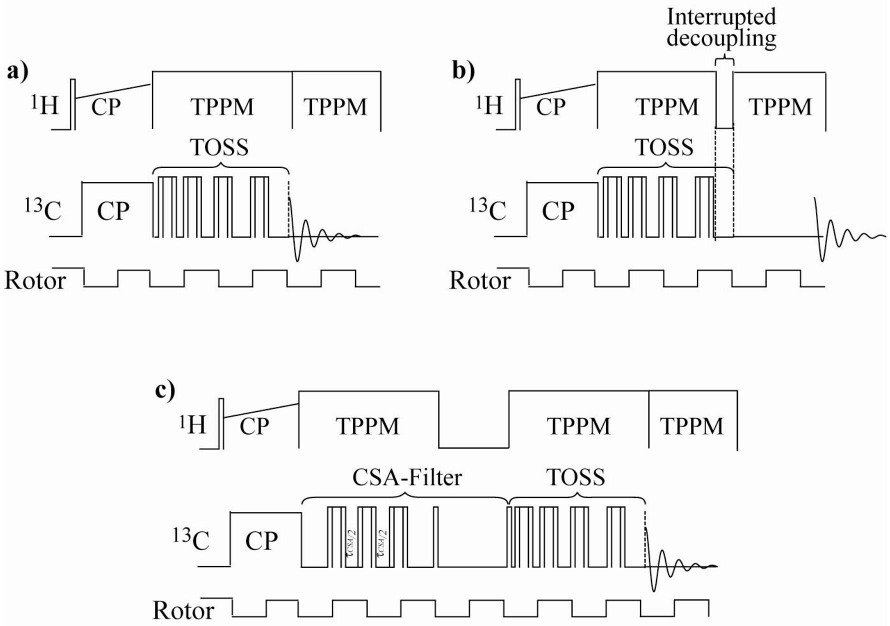

Figure A1. NMR pulse sequences used in the study of pretreated sugarcane bagasse: (a) ramped CPMAS-TOSS experiment; **(b)** ramped CPMAS-TOSS experiment with interrupted decoupling for dipolar dephasing; (**c)** CSA filtered ramped CPMAS-TOSS.

References of the additional file 1:

1. Knicker H, Totsche KU, Almendros G, González-Vila FJ: **Condensation degree of burnt peat and plant residues and the reliability of solid-state VACP MAS ^{13}C NMR spectra obtained from pyrogenic humic material.** *Org Geochem* 2005, **36**:1359-1377.

2. Dixon WT, Schaefer J, Sefcik MD, Stejskal EO, Mckay RA: **Total Suppression of Sidebands in CPMAS C-13 NMR.** *J Magn Reson* 1982, **49**: 341-345.

3. Novotny EH, Hayes MHB, deAzevedo ER, Bonagamba TJ: **Characterisation of Black Carbon-rich samples by ^{13}C solid-state nuclear magnetic resonance**. *Naturwissenschaften* 2006, **93**:447-450.

4. Mao JD, Schmidt-Rohr K: **Accurate quantification of aromaticity and nonprotonated aromatic carbon fraction in natural organic matter by ^{13}C solid-state nuclear magnetic resonance.** *Environ Sci Technol* 2004, **38**:2680-2684.

5. Novotny EH, deAzevedo ER, Bonagamba TJ, Cunha TJF, Madari BE, Benites VDM, Hayes MHB: **Studies of the Compositions of Humic Acids from Amazonian Dark Earth Soils.** *Environ Sci Technol* 2007, **41**:400-405.
