# Supplementary material for: Chemical and morphological characterization of sugarcane bagasse submitted to a delignification process for enhanced enzymatic digestibility
Source: Biotechnol Biofuels. 2011 Nov 28;4:54. doi: 10.1186/1754-6834-4-54 (PMC3377919; doi:10.1186/1754-6834-4-54)
Supplement: Additional file 2 — DRIFT experiments. This file contains results obtained on sugarcane bagasse samples using diffuse reflectance Fourier transformed infrared (DRIFT) spectroscopy (pdf). [file 1754-6834-4-54-S2.PDF]

Supplementary information for the paper:

Chemical and morphological characterization of sugarcane bagasse submitted to delignification process for enhanced enzymatic digestibility

Authors: Camila Alves Rezende; Marisa Aparecida de Lima; Priscila Maziero, Eduardo Ribeiro deAzevedo; Wanius Garcia; Igor Polikarpov.

Additional information 2: DRIFT experiments

Diffuse Reflectance Fourier Transformed Infrared (DRIFT) spectroscopy is an excellent tool for chemical analysis of lignocellulosic biomass, since it is non-destructive and requires minimal sample preparation. It is also a fast and direct technique that has been employed to reduce base-line errors caused by light scattering [1].

DRIFT spectra of untreated sugarcane bagasse and of samples treated with different NaOH concentrations are shown in Figure A1. Table A1 shows a summary of the main peaks (numbered from 1 to 14 on the untreated bagasse spectrum in Figure A1) and their respective assignments, whose determination was based on published data [2-5]. Due to biomass complexity, most of the spectral bands have contributions from its three major components (lignin, cellulose and hemicelluloses).

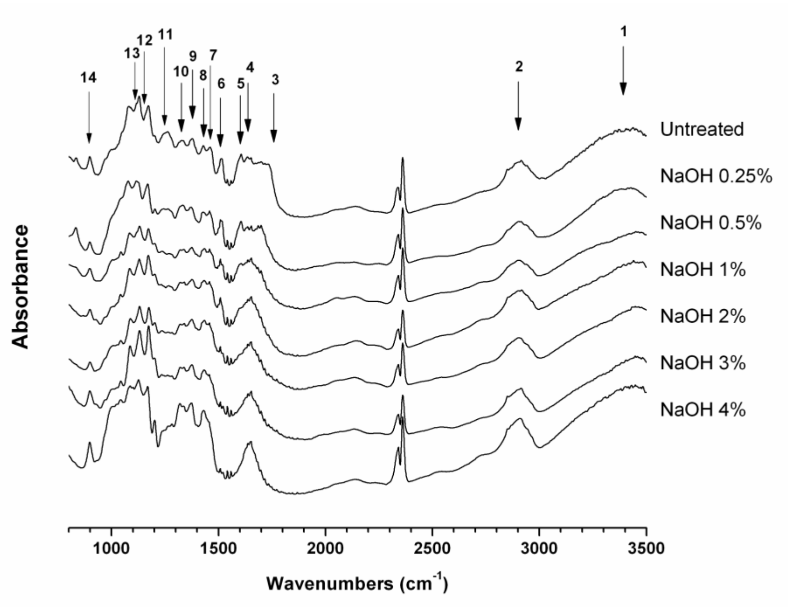

Figure A1. DRIFT spectra of untreated sugarcane bagasse and samples which underwent pretreatment with increasing NaOH concentrations.

Table A1. Assignments of DRIFT peaks 1 to 14 indicated on the untreated bagasse spectrum in Figure A1 [2-34].

Peak number	Peak (cm^{-1})	Assignment
1	3435	stretching O-H asymmetric
2	2917	stretching C-H
3	1729	C=O band (polysaccharides, hemicellulose)
4	1648	O-H and conjugated C-O
5	1596	aromatic skeletal vibration plus C=O stretch
6	1510	aromatic skeletal vibration in lignin (C=C)
7	1460	C-H deformation in lignin and carbohydrates
8	1425	C-H deformation in lignin and carbohydrates
9	1375	C-H deformation in cellulose and hemicellulose
10	1325	C-H vibration in cellulose and C-O vibration in syringyl derivatives
11	1263	guaiacyl ring breathing (softwood lignin), C-O stretch in lignin and C-O linkage in guaiacyl aromatic methoxyl groups
12	1170	C-O-C vibration in cellulose and hemicellulose
13	1124	aromatic skeletal and C-O stretch
14	902	C-H deformation in cellulose

DRIFT spectra in Figure A1 reveal major changes in peaks 3, 4, 5, 6 and 11 (1729, 1648, 1596, 1510 and 1263 cm^{-1}, respectively) as a consequence of the alkaline treatment. Peak 3, characteristic of hemicellulose groups, such as acetyl and uronic esters, is only present in the untreated sample. DRIFT spectra also shows significant decrease of intensity on the region of peak 4, related to ketone groups from lignin involved in intramolecular hydrogen bonds (absorbed O-H and conjugated C-O). This decrease can be explained by the degradation of phenyl propane units in the lignin structure [4]. Significant intensity decrease was also observed in peak 5, which corresponds to aromatic skeletal ring vibration and carbonyl groups. Peak 6 at 1510 cm^{-1} is the only "pure" band related to the aromatic moieties present in lignin and it is frequently used as a reference [5]. This peak gradually disappears as sodium hydroxide concentration increases. Finally, lignin removal is also indicated by the relevant intensity decrease in peak 11, which corresponds to characteristic signals of softwood lignin (guaiacyl derivatives), the major lignin component of sugarcane. Lignin removal and cellulose enrichment due to alkaline pretreatments are also evidenced by calculating the ratio between the area of the cellulose characteristic peak at 902 cm^{-1} (peak 14) and that of the lignin peak at 1510 cm^{-1} (peak 6). Ratios obtained from DRIFT data (I_{902}/I_{1510}) for all the samples are summarized in Table A2 and compared to the ratios of cellulose/lignin weights (w_{cel}/w_{lig}) in each sample. The cellulose/lignin weight ratio is obtained from HPLC and UV-VIS data and takes into account the cellulose loss during pretreatment. It is possible to observe that ratios calculated for cellulose and lignin peak areas increased, following the increase in the cellulose/lignin weight ratio (w_{cel}/w_{lig}). Taken together, these two ratios confirm the efficiency of the employed pretreatment method for removing lignin and producing cellulose-rich samples.

Table A2: Ratios for cellulose and lignin peak areas (I_{902}/I_{1510}) obtained by DRIFT, and for cellulose and lignin weight (w_{cel}/w_{lig}) determined by the total acid hydrolysis method.

Bagasse samples	Relative area of cellulose and lignin peaks (I_{902}/I_{1510})	Relative amount of cellulose and lignin (w_{cel}/w_{lig})
Untreated	1.05	1.59
NaOH 0.25%	1.08	2.38
NaOH 0.5%	1.98	2.49
NaOH 1.0%	4.03	5.38
NaOH 2.0%	4.43	6.10
NaOH 3.0%	5.42	6.35
NaOH 4.0%	6.65	5.91

Methods

DRIFT spectra were obtained at room temperature from sugarcane bagasse samples before and after pretreatment steps. Analyses were performed using a Varian FTIR spectrometer at a wavenumber setting ranging from 400 to 4000 cm^{-1}. Five measures were recorded for each pretreated sample, and 64 scans were accumulated for all spectra at a 4 cm^{-1}resolution.

References for the additional file 2:

1. Gonçalves AR, Moriya RY, Oliveira LRM, Saad MBW: **Xylanase recycling for the economical biobleaching of sugarcane bagasse and straw pulps.** *Enzyme Microb Technol* 2008, **43**:157-163.

2. Pandey KK, Pitman AJ: **FTIR studies of the changes in wood chemistry following decay by brown-rot and white-rot fungi.** *Int Biodeter Biodegradation* 2003, **52**:151-160.

3. Pandey KK, Nagveni HC: **Rapid characterization of brown and white rot degraded chir pine and rubberwood by FTIR spectroscopy.** *Holz Roh Werkst* 2007, **65**:477-481.
4. Guo GL, Hsu DC, Chen WH, Chen WH, Hwang WS: **Characterization of enzymatic saccharification for acid-pretreated lignocellulosic materials with different lignin composition**. *Enzyme Microb Technol* 2009, **45**:80-87.
5. Ferraz A, Baeza J, Rodriguez J, Freer J: **Estimating the chemical composition of biodegraded pine and eucalyptus wood by DRIFT spectroscopy and multivariate analysis.** *Biores Technol* 2000, **74**: 201-212.
